# Robust Acoustic Imaging Based on Bregman Iteration and Fast Iterative Shrinkage-Thresholding Algorithm

**DOI:** 10.3390/s20247298

**Published:** 2020-12-18

**Authors:** Linsen Huang, Shaoyu Song, Zhongming Xu, Zhifei Zhang, Yansong He

**Affiliations:** School of Automotive Engineering, Chongqing University, 174 Shazhengjie, Chongqing 400044, China; 20183201008@cqu.edu.cn (L.H.); 20153202019t@cqu.edu.cn (S.S.); z.zhang@cqu.edu.cn (Z.Z.); hys68@cqu.edu.cn (Y.H.)

**Keywords:** acoustic imaging, beamforming, Bregman iteration method, near-field acoustic holography, sparse representation

## Abstract

The acoustic imaging (AI) technique could map the position and the strength of the sound source via the signal processing of the microphone array. Conventional methods, including far-field beamforming (BF) and near-field acoustic holography (NAH), are limited to the frequency range of measured objects. A method called Bregman iteration based acoustic imaging (BI-AI) is proposed to enhance the performance of the two-dimensional acoustic imaging in the far-field and near-field measurements. For the large-scale $\ell_{1}$ norm problem, Bregman iteration (BI) acquires the sparse solution; the fast iterative shrinkage-thresholding algorithm (FISTA) solves each sub-problem. The interpolating wavelet method extracts the information about sources and refines the computational grid to underpin BI-AI in the low-frequency range. The capabilities of the proposed method were validated by the comparison between some tried-and-tested methods processing simulated and experimental data. The results showed that BI-AI separates the coherent sources well in the low-frequency range compared with wideband acoustical holography (WBH); BI-AI estimates better strength and reduces the width of main lobe compared with $\ell_{1}$ generalized inverse beamforming ($\ell_{1}$-GIB).

## 1. Introduction

As a powerful tool for acoustic visualization, the acoustic imaging (AI) characterizes the spatial field generated by the sound sources and details the performance and mechanisms of sources in the transportation, machinery industry as well as biomedical applications [1,2,3]. Two standard methods of acoustic imaging are far-field beamforming (BF) [4,5] and near-field acoustical holography (NAH) [6,7,8]. Both of them calculate the sound field data from an array of multiple microphone sensors on the holography plane via some data-processing reconstruction algorithms. They have an edge on identifying sound sources and reconstructing the three-dimensional sound field visually. In this work, we concentrate on the solving of the linear system, which is the model for many inverses based on beamforming and near-field acoustical holography techniques.

The resolution quality and the strength estimation evaluate the performance of BF while the reconstruction accuracy grades the performance of NAH. In the field of BF, conventional frequency-domain beamforming suffers from poor spatial resolution quality and struggles to quantify the sources in the low-frequency range [9]. Schmidt [10] proposed the multiple signal classification (MUSIC) to enhance the resolution quality on a priori source number, but this method failed to estimate the source power. Stoica et al. [11] stated the robust Capon Beamforming to calculate the amount of diagonal loading based on the uncertainty of the steering vector, with the array covariance matrix required as prior. Wang et al. [12] ran an expectation–maximization algorithm based on beamforming, which is called iterative beamforming, to estimate the location and the power of sources. In the field of NAH, the Fourier transform based on NAH is established on the 2D decomposition of hologram pressure into equidistant spatial Fourier basis in *k*-space [7]. To meet the Nyquist–Shannon sampling theorem and reduce the spatial wraparound error, many microphones are required in the measurements in the medium-frequency and high-frequency range. For nonplanar sources, some methods were proposed, such as inverse boundary element methods (IBEM) [13], Helmholtz equation-least squares (HELS) [14], statistically optimized near-field acoustical holography (SONAH) [15], and the equivalent source method-based NAH [16,17]. Taken together, beamforming gains unsatisfied resolution quality and strength estimation in the low-frequency range; near-field acoustical holography underperforms in the medium-frequency and high-frequency range.

In the community of signal processing, the compressive sensing (CS) [18,19] exploits the sparsity of data to recover an underlying signal with measurements fewer than the Nyquist sampling rate. CS could solve a convex optimization problem and reconstruct the spatial compact/sparse sources with super-resolution. As the core of CS, the $\ell_{1}$ norm sense has been used in the field of acoustic imaging such as $\ell_{1}$ norm generalized inverse beamforming ($\ell_{1}$-GIB) [20], wideband acoustical holography (WBH) [21], and sparse regularization [22,23,24], to name only a few.

The goal of this work is to assess the depressed performance of WBH on separating the coherent sources in the low-frequency and medium-frequency range. To that end, accomplished by exploiting the sparse property of signal and incoherent sampling property, the far-field beamforming and near-field acoustical holography in the frame of CS are reformulated. The Bregman iteration is a well established method for the solution of $\ell_{1}$-regularized optimization problems. It has been successfully applied not only in compressed sensing but also in different fields, such as image analysis [25,26], matrix rank minimization [27], and finance [28,29]. The Bregman iteration (BI) [30] method is introduced to solve the problem in the linear system. The problem is decomposed into a sequence of sub-problems without restrictive conditions to simplify the calculation. For each sub-problem of $\ell_{1}$ norm minimization, the fast iterative shrinkage-thresholding algorithm (FISTA) [31,32] is adopted. Meanwhile, the interpolating wavelet method strengthens the performance of the proposed method in the low-frequency range.

The outline of the rest of the sections is as follows. Section 2 theorizes the numerical model. Section 3 simulates numerically and compares with some tried-and-tested methods of sound field reconstruction and noise source identification to benchmark the performance of the proposed method. Section 4 processes experimental data via these above-mentioned methods to check their ability. Section 5 concludes this paper.

## 2. Bregman Iteration Based Acoustic Imaging

### 2.1. Bregman Iteration with FISTA

**Figure 1:**
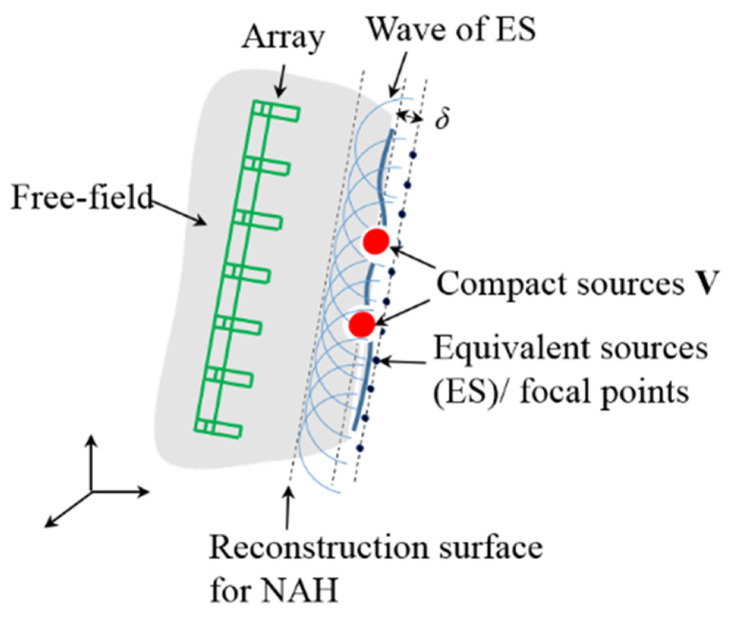
The diagrammatic sketch of the source-receiver model for acoustic imaging.

The sound field of time-harmonic compact radiators V is simultaneously measured by an array of M microphones located at $r_{m}$, m = 1, 2, *…*, M (Figure 1). The exterior sound pressure at $r_{m}$ could be formulated by the superposition principle as Equation (1) expressed:

(1) $$p\left(r_{m}\right)=j\rho_{0}\omega \int_{S}q\left(r_{0}\right)g\left(r_{m},r_{0}\right)dS\left(r_{0}\right)$$

where $\rho_{0}$ and ω are the medium density and the angular frequency, respectively; $j=\sqrt{-1}$. $q(r_{0})$ is the source strength at $r_{0}$ on S; $g(r_{m},r_{0})$ is the propagation relation between positions $r_{m}$ and $r_{0}$.

Expanding the term $j\rho_{0}\omega q\left(r_{0}\right)$ in Equation (1) with N pulse basis functions (a.k.a. elementary wave functions), Equation (1) could be rewritten as Equation (2):

(2) p=Gq

where $p={\left[p\left(r_{1}\right),\ldots ,p\left(r_{M}\right)\right]}^{T}$ is the hologram pressure vector, $q=j\rho_{0}\omega {\left[q\left(w_{1}\right),\ldots ,q\left(w_{N}\right)\right]}^{T}$ symbolizes the strength information at the source position $w_{n}$ (n = 1, 2, …, N), $G_{\mathrm{mn}}=\exp\left(-{\mathrm{jkr}}_{\mathrm{mn}}\right)/\left(4\pi r_{\mathrm{mn}}\right)$ is the propagation matrix associating measured pressure with source strength, $r_{\mathrm{mn}}=\left|r_{m}-w_{n}\right|$. Conventional equivalent source method (CESM) [8,33] adopts the Tikhonov regularization method in the solving of inverse problems; it determines the regularization parameter via the L-curve method. CESM solves Equation (2) in the least square sense, but it tends to give blur and smooth equivalent sources strength distribution, whereas the most acoustic sources are sparse to some extent. The $\ell_{2}$ norm problem solved by CESM could be recast as the $\ell_{1}$ norm minimization model by sparsity-promoting [34].

(3) $$\min_{q}{∥q∥}_{1}\;\;s.t.\;p=\mathrm{Gq}$$

Equation (3) could be recast to solve an unconstrained problem:

(4) $$\min_{q}{∥p-Gq∥}_{2}^{2}+\mu {∥q∥}_{1}$$

The sparsity of the solution could be acquired by Equation (4). However, it is difficult to determine the parameter μ, which is chosen empirically. Pereira et al. [35] optimized a method of choosing the regularization parameter.

In this present paper, we introduce the Bregman iteration to derive the solution to Equation (3). The constraint of the radiation model fails to be rigorously satisfied because of the noise in the measured data. Equation (3) could be recast to:

(5) $$\min_{q}{∥q∥}_{1}\;\;s.t.\;{∥p-\mathrm{Gq}∥}_{2}\leq \varepsilon$$

where $\varepsilon ={∥p∥}_{2}10^{-\mathrm{SNR}/20}$ is the noise level. Instead of solving Equation (4) or Equation (5) once [36], the Bregman iteration constructs a sequence of unconstrained sub-problems such that each solution of the sub-problems is $q_{K}$ with K the index of the solution of the sub-problems. To approximate the actual cases, a discrepancy stopping criterion ${∥Gq_{K}-p∥}_{2}\leq \varepsilon$ is adopted. The sequence of sub-problems herein is in Equation (6):

(6) $$q_{K+1}=\arg\min_{q}D_{J}^{b_{K}}\left(q,q_{K}\right)+\frac{\lambda }{2}{∥Gq-p∥}_{2}^{2}$$

where $D_{J}^{b_{K}}\left(q,q_{K}\right)=J\left(q\right)-J\left(q_{K}\right)-\langle b_{K},q-q_{K}\rangle$ is the Bregman distance between q and the obtained source distribution of previous sub-problem $q_{K}$, and $\langle b_{K},q-q_{K}\rangle$ is the inner product of differential $b_{K}$ and $q-q_{K}$, J(q) is a convex source energy function (in this case, $J\left(q\right)={∥q∥}_{1}$, $b_{K}=\sup\left(q_{K}\right)$ is the sub-differential of function J(q) at $q_{K}$, and constant λ is an iterative regularization parameter which makes a trade-off between the Bregman distance and the $\ell_{2}$ norm term. The initial values are $q_{0}=0$ and sound pressure $p_{0}=0$. Since $0\in \partial \left(D_{J}^{b_{K}}\left(q,q_{K}\right)+\frac{\lambda }{2}{∥Gq-p∥}_{2}^{2}\right)$, we update the solution in the sense of Bregman distance $b_{K+1}=b_{K}-\lambda G^{H}\left(Gq_{K}-p\right)$. The Bregman iteration could be expressed in Equations (7) and (8):

(7) $$q_{K+1}=\arg\min_{q}{∥q∥}_{1}-\langle p_{K},q-q_{K}\rangle +\frac{\lambda }{2}{∥Gq-p∥}_{2}^{2}$$

(8) $$b_{K+1}=b_{K}-\lambda G^{H}\left(Gq_{K+1}-p\right)$$

This iteration strategy [30,37] could be equivalent to Equations (9) and (10) because of the linearity of the constraints:

(9) $$q_{K+1}=\arg\min_{q}{∥q∥}_{1}+\frac{\lambda }{2}{∥Gq-p_{K}∥}_{2}^{2}$$

(10) $$p_{K+1}=p_{K}+p-Gq_{K}$$

The iterative shrinkage-thresholding algorithm (ISTA) could efficiently solve Equation (9) as Equation (11) expressed:

(11) $${\tilde{q}}_{k+1}=S_{t_{k}/\lambda }\left({\tilde{q}}_{k}-t_{k}G^{H}\left(G{\tilde{q}}_{k}-p_{K}\right)\right)$$

where the step length $t_{k}\in \left(0,2/{∥GG^{H}∥}_{2}\right)$ could guarantee the convergence [30,38]; ${\tilde{q}}_{k}$ is the source strength for sub-problems with k the index of the inner loop. In this paper, $t_{k}$ is set as $1.99/{∥GG^{H}∥}_{2}$. Equation (12) represents the thresholding operator $S_{\alpha }$:

(12) $$S_{\alpha }{\left(x\right)}_{i}={\left(\left|x_{i}\right|-\alpha \right)}_{+}\mathrm{sign}\left(x_{i}\right)$$

where the operator ${\left(\left|x_{i}\right|-\alpha \right)}_{+}=\max\left\{0,\left|x_{i}\right|-\alpha \right\}$. FISTA is a first-order method for minimizing objective function given by the summation of smooth and non-smooth terms. Beck et al. [39] gathered the previous two-step information as the extrapolation on the results and drew a variant version of the iterative shrinkage-thresholding algorithm (FISTA):

(13) $${\tilde{q}}_{k}=S_{T_{k}/\lambda }\left(y_{k}-T_{k}G^{H}\left(Gy_{k}-p_{K}\right)\right)$$

(14) $$T_{k+1}=\frac{1+\sqrt{1+4T_{k}^{2}}}{2}$$

(15) $$y_{k+1}={\tilde{q}}_{k}+\left(\frac{T_{k}-1}{T_{k+1}}\right)\left({\tilde{q}}_{k}-{\tilde{q}}_{k-1}\right)$$

where $T_{1}=1$. The phase angle of the source distribution is updated synchronously with the acceleration process of Equation (15). In the outer-iteration (Equations (9) and (10)), the noise produced by the previous iteration could be filtered.

The initial values of the original Bregman iteration are named “cold start” in literature [40]. Below 3000 Hz, it is more suitable for “cold start”. In this present paper, the output of conventional beamforming is designed as input when the frequency is above 3000 Hz.

(16) $$q_{1}=G^{H}p/M,p_{1}=p_{0}+p-Gq_{0}$$

It shows that this kind of “warm start” could speed up the convergence to some degree in our simulation. The classical method of adding a penalty function to simplify Equation (5) is to carry out a fixed regularization parameter tuned by hand. It is a workable way for convergence assurance to construct an increasing sequence of penalty parameter terms, $\lambda_{1}<\lambda_{2}<\cdots <\lambda_{M}$. To enforce the residue ${∥Gq_{k}-p∥}_{2}\approx 0$, $\lambda_{M}$ should be chosen large enough but a large value of $\lambda_{M}$ would make the problem difficult to solve. In the iterative strategy of BI, Equation (9) is proved to converge quickly with a constant regularization parameter λ [39]. We suggest the parameter λ as

(17) $$\lambda =\frac{\rho }{{∥G^{H}p∥}_{\infty }}$$

where ρ affects the convergence speed of BI. A small ρ slows down the convergence. In this study, ρ is suggested to be 0.1 according to the results of many simulations and experiments.

In the inevitable presence of noise, ${∥Gq_{k}-p∥}_{2}\leq \varepsilon$ is a discrepancy stopping criterion to make a trade-off between sparsity prior and data perturbation. For a larger ε, a sparser representation of sources could be achieved, whereas the reconstruction accuracy may decrease. The problem could be expressed as p=Gq+n. The term $\varepsilon ={∥n∥}_{2}$ is defined as a function of the noise strength (including electronic noise, background noise, and so on) in which background noise is assumed to be white noise and the main part of overall noise. Owing to the convergence of Bregman iteration, the distance measured between the true pressures and the iteration, decreases monotonically. ε is determined by SNR in our study; another method to determine the value of ε could be found in reference [22] where $\varepsilon ={\alpha ∥p∥}_{2}$ with α = 20∼30% suggested.

During the solving process of sub-problems, FISTA computes the sub-differential of Equation (9). The stopping criteria of FISTA are shown in Equation (18):

(18) $${∥G^{H}\left(G{\tilde{q}}_{k}-p_{K}\right)∥}_{2}\leq \delta {∥G^{H}\left(G{\tilde{q}}_{1}-p_{1}\right)∥}_{2}\;\mathrm{or}\;k\geq k_{\max\;}$$

where δ=0.001. If the $\ell_{2}$ distance between sub-differential of Equation (9) and zero comes to the tolerance or the number of iterations k reaches the maximum, the algorithm will refresh $p_{k+1}$ and compute the next sub-problem on the condition that ${∥Gq_{K}-p∥}_{2}\leq \varepsilon$ is not satisfied.

### 2.2. Refining the Computational Grid via the Wavelet Method

The basis of Fourier based NAH is evenly localized in the wave-number space. The scanning points of BF are symmetrical. Few of them localize in physics as they have global support, which leads to the position deviation of the low-frequency source. However, the base’s functions of wavelets are localized in the physical space and the wave-number space both. To extract the spatial information to design the base function, a method of grid refining works in the low-frequency range. The source map Ω generated by conventional acoustic imaging methods contains source information. To deploy the information as prior and prevent spurious sources distribution in the non-source region, the interpolating wavelet method [41] implements the multi-resolution analysis and extracts sources distribution. We briefly introduce the one-dimensional case of wavelets which are located at the points of the nested grid of Ω. The 2D wavelets expand the index in one dimension to the matrix.

A scalar function f(x), which is the outputs of the statistically optimal array processing (SOAP) in this case, on the discrete nested grids could be represented by wavelet basis functions on J levels of resolution as in Equation (19):

(19) $$f\left(x\right)=\sum_{k\in K^{\circ }}c_{k^{0}}\phi_{k^{0}}\left(x\right)+\sum_{j=0}^{J-1}\sum_{l\in L^{j}}d_{l}^{j}\psi_{l}^{j}\left(x\right)$$

where *x* is the grid point, *j* is the level index, $c_{k}^{0}$, and $d_{l}^{j}$ are the scaling coefficients of the lowest level and the wavelet coefficients on level *j*, $\phi_{k}^{0}\left(x\right)$ and $\psi_{l}^{j}\left(x\right)$ are scaling functions and wavelets at physical position of point *x*, and $L^{j}$ and $\kappa^{0}$ are the index sets of wavelets and scaling functions, respectively. The grid points $x_{k}^{j}$ on jth resolution form a layer of nested grids as:

(20) $$G^{j}:=\left\{x_{k}^{j}\in \Omega |x_{k}^{j}=x_{2k}^{j+1},k\in Z,j\leq J-1\right\}$$

where condition $x_{k}^{j}=x_{2k}^{j+1}$ guarantees $G^{j}\subset G^{j+1}$, namely, the point in each level belongs to its adjacent higher grid level. *k* is the successive index of the grid point. The f(x) could be approximately acquired by discarding the wavelets with relatively small wavelet coefficients. Therefore, the decomposed form of f(x) can consist of two parts as Equation (21) shows:

(21) $$f\left(x\right)=f_{\varepsilon }^{J}\left(x\right)+R_{\varepsilon }^{J}\left(x\right)$$

where

(22) $$f_{\varepsilon }^{J}\left(x\right)=\sum_{k\in K^{\circ }}c_{k^{0}}\phi_{k^{0}}\left(x\right)+\sum_{j=0}^{J-1}\sum_{l\in L^{j},\left|d_{l}^{j}\right|\geq \varepsilon }d_{l}^{j}\psi_{l}^{j}\left(x\right)$$

(23) $$R_{\varepsilon }^{J}\left(x\right)=\sum_{j=0}^{J-1}\sum_{l\in L^{j}\left|d_{l}^{j}\right|<\varepsilon }d_{l}^{j}\psi_{l}^{j}\left(x\right)$$

By decomposing, the point set on each level could be expressed as:

(24) $$G^{j}:=G_{\geq }^{j}\cup G_{<}^{j}$$

where

(25) $$G_{\geq }^{j}:=\left\{x_{k}^{j}\in G^{j}\;s.t.\;\left|d_{l}^{j}\right|\geq \varepsilon \right\}\;\;\mathrm{and}\;\;G_{<}^{j}:=\left\{x_{k}^{j}\in G^{j}\;s.t.\;\left|d_{l}^{j}\right|<\varepsilon \right\}$$

The set of essential points $G_{\geq }$ could be corrected together as:

(26) $$G_{\geq }:=\cup_{j=0}^{J}G_{\geq }^{j}$$

The set $G^{0}$ of coarsest nested grids is always included in $G_{\geq }$ because it is only related to the scaling functions. The sparse wavelet representation of a source map is realized by discarding the small wavelets. Consequently, the essential points which represent the source distribution could be obtained. The coefficients $d_{l}^{j}$ of interpolating wavelets could be fast calculated by hierarchical applying interpolating on level j−1 to the difference set between *j* resolution and j−1 resolution. As previously described [42,43,44], $d_{l}^{j}$ is derived by subtracting interpolating value from the true value in level *J*. The optimized computational grids could be formed via extracting essential points with the wavelets method.

## 3. Simulations

This section explored the proposed method by numerical simulations. A rectangular plane array of M=81 microphone elements uniformly spaced with the dimensions of 0.8 m × 0.8 m was used to perform focusing and reconstruct the sound field. The center of the array was settled at the origin of coordinate. The single monopole source and two coherent monopole sources were tested, respectively. The single source was arranged at the center of the interested plane while two coherent sources were located at (−0.4, 0) m and (0.4, 0) m on this plane. The number of scanning points (i.e., the equivalent sources) was set as 61 × 61, which were uniformly arranged in an area of 1.2 m × 1.2 m with 0.02 m spacing.

### 3.1. BI-AI in the Near-Field Measurements

This numerical simulation subsection reveals the results of sound field reconstruction via BI-AI in the near-field measurements. The proposed method was compared with two tried-and-tested methods including the conventional equivalent source method (CESM) and the wideband acoustical holography (WBH). The geometry of the array is the aforementioned rectangular array as well. The SNR is 30 dB. The holographic distance is 0.2 m and the reconstruction surface is 0.07 m away from the sound source plane. In the case of sparse sources, the retreated distance is 0.01 m, and the same setting was recorded in WBH [21]. To quantitatively assess the reconstruction performance of BI-AI, Equation (27) defined the relative error of reconstructed pressure:

(27) $$re\left(w\right)=\frac{{∥p_{t}\left(w\right)-p_{r}\left(w\right)∥}_{2}}{{∥p_{t}\left(w\right)∥}_{2}}\times 100\%$$

where $p_{t}$ and $p_{r}$ is the true and reconstructed pressure matrix (or vectors) on the targeted plane, respectively.

**Figure 2:**
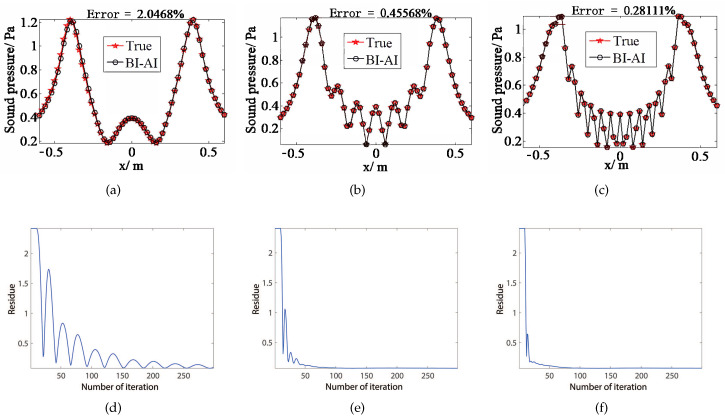
The sound field reconstruction with BI-AI at the frequencies of 500 Hz (**a**,**d**), 1500 Hz (**b**,**e**), and 3000 Hz (**c**,**f**). (**a**–**c**) are the reconstruction pressure along the *x*-axis.

Figure 2 indicates the reconstruction results of 500 Hz, 1500 Hz, and 3000 Hz sources via BI-AI. The iteration of the outer loop of BI-AI stops if the residue reaches $\varepsilon =10^{-15/20}{∥p∥}_{2}$. The sound fields of sources are successfully reconstructed in Figure 2a–c in which the relative error on the *x*-axis are 2.05%, 0.46%, and 0.28%, respectively. BI-AI delivers visible deviation from the true value at the frequency of 500 Hz (Figure 2a). By contrast, the sound pressure generated by equivalent source fits the true field well at the frequencies of 1500 Hz and 3000 Hz (Figure 2b,c). As for the convergence of the proposed method, it is observed by extensive simulations that BI-AI is prone to converge in dozens of steps.

**Figure 3:**
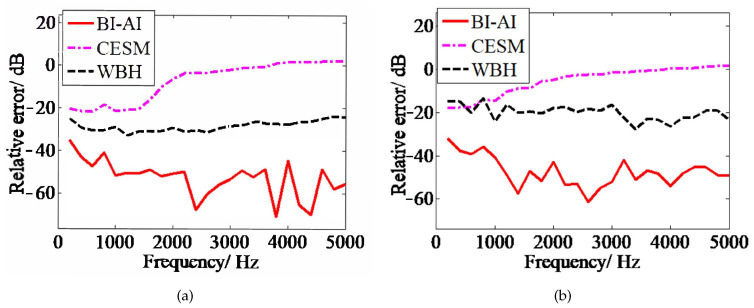
The relative error of reconstructed sound field for three acoustical holography methods including CESM, WBH, and BI-AI at frequencies of 200 Hz to 5000 Hz: (**a**) monopole sound source with unitary strength; (**b**) two coherent, in-phase monopole sound sources with strength of 1 and 0.5.

Next, we display the relative error (mean of 10 simulations) of sound field reconstruction in the logarithmic scale in Figure 3 to highlight the minor differences, namely, $20\times \log_{10}\left(re\right)$. It delivers that the relative error of CESM is larger in the high-frequency range, especially larger than 0 dB above 3600 Hz (Figure 3a) and 4000 Hz (Figure 3b). The relative errors of WBH are stable in the interested frequency range, which are about −30 dB and −20 dB, respectively. The relative error of BI-AI is basically below −40 dB except for 200 Hz for single monopole and below 1000 Hz for coherent monopoles. The relative error of the sound field reconstruction by BI-AI is smaller than that of CESM and WBH in the interested frequency range.

### 3.2. Source Detection in the Far-Field Measurements

The sources are located at 1 m away from the array plane for the far-field measurements. The proposed method was compared with the existing detection methods including the statistically optimal array processing (SOAP) and the $\ell_{1}$ generalized inverse beamforming ($\ell_{1}$-GIB). The regularization parameter in SOAP and $\ell_{1}$-GIB was 0.1∼10% of the maximum eigenvalue of $AA^{H}$ and $A\left(W\right)A^{H}$. Here, W is the weighting coefficient matrix. For $\ell_{1}$-GIB, the reduction factor is 0.9. Additionally, 10 steps of iterations without reducing the scanning points stabilized the calculation.

**Figure 4:**
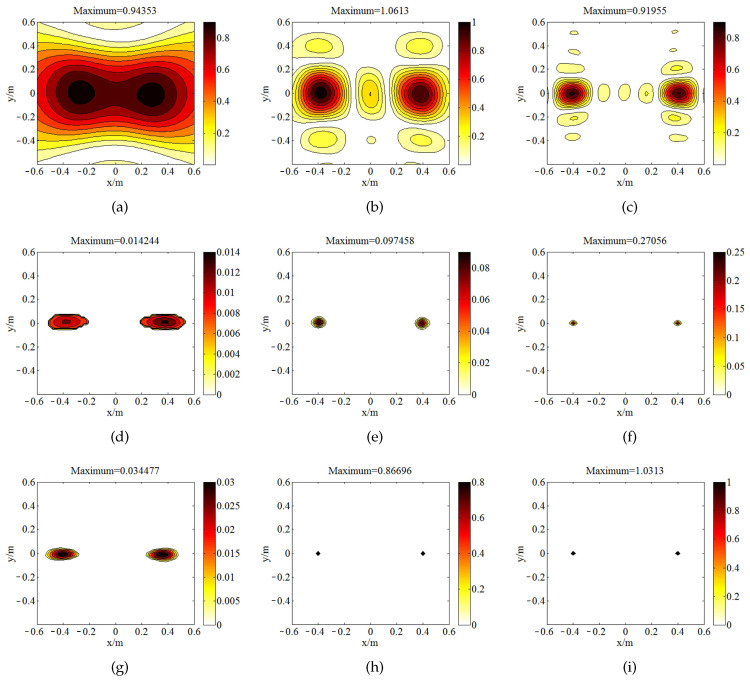
The comparison of sources maps (with unit of $m^{4}/s^{2}$) in the simulation of focusing: (**a**) SOAP, 500 Hz; (**b**) SOAP, 1500 Hz; (**c**) SOAP, 3000 Hz; (**d**) $\ell_{1}$-GIB, 500 Hz; (**e**) $\ell_{1}$-GIB, 1500 Hz; (**f**) $\ell_{1}$-GIB, 3000 Hz; (**g**) BI-AI with 20 inner iterations, 500 Hz; (**h**) BI-AI with 20 inner iterations, 1500 Hz; (**i**) BI-AI with 20 inner iterations, 3000 Hz.

Figure 4 is the source map of two coherent monopole sources (with unitary strength) generated by the three above-mentioned approaches. All of them detect the coherent sources at the correct position except Figure 4a. The maximal value acquired by SOAP is close to the true value. Nevertheless, the strength in the non-source region fails to indicate the radiation characteristics of the source plane. SOAP narrows the main lobes in the high-frequency range. In contrast to the inferior resolution quality in SOAP outputs, $\ell_{1}$-GIB and the proposed method delivers narrower main lobes, with the peak value underestimated. At the frequencies of 1500 Hz and 3000 Hz, the source maps of BI-AI are the sparsest among these methods. BI-AI (Figure 4g–i) deliveries larger maximum peak value compared with $\ell_{1}$-GIB (Figure 4d–f) at the frequencies of 500 Hz, 1500 Hz, and 3000 Hz, respectively.

**Figure 5:**
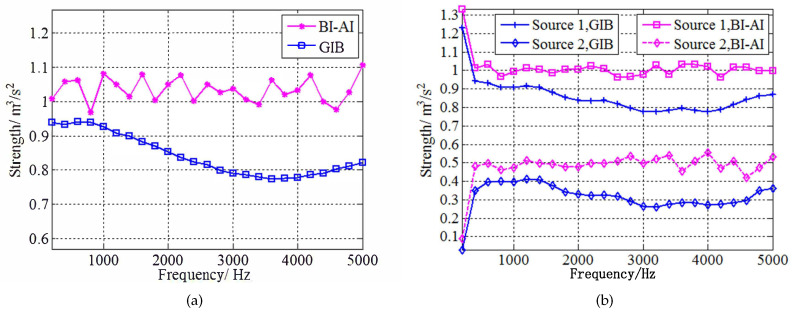
The strength estimation varying radiation frequency (20 inner iterations, 300 outer iteration). (**a**) one unitary monopole; (**b**) two coherent monopole sound sources, the strength of sources is 1 and 0.5, respectively.

On top of that, we evaluate quantitatively the performance of the three mentioned methods by the accuracy of the strength estimation and the resolution quality. The estimated radiation strength is defined as the summation of identified strength on the source plane; the resolution quality is characterized by the beam area within −12 dB down from the peak value of the main lobe. Figure 5 indicates the estimated strength in radiant surface versus the source frequency for the single source and the coherent sources, respectively. For the coherent sources, the strength of one source is 1 while the other is 0.5. As SOAP took on the meaningless solution to the non-source zone as Figure 4 depicted, the strength estimation of SOAP was not summed for reference. The strength curve of $\ell_{1}$-GIB decreases in the medium-frequency and high-frequency range as per references [20,45]. By comparison, BI-AI gives robust estimation from 200 Hz to 5000 Hz in Figure 5a, and 400 Hz to 5000 Hz in Figure 5b. The results reveal that BI-AI outweighs $\ell_{1}$-GIB in both cases of single and coherent sources in strength estimation. BI-AI improves the quality of strength estimation and allows the detection of a somewhat wake source without contaminating the maps with side lobes. It suggests that the noise in solution is removed by iterative shrinkage in the solving of sub-problem.

**Figure 6:**
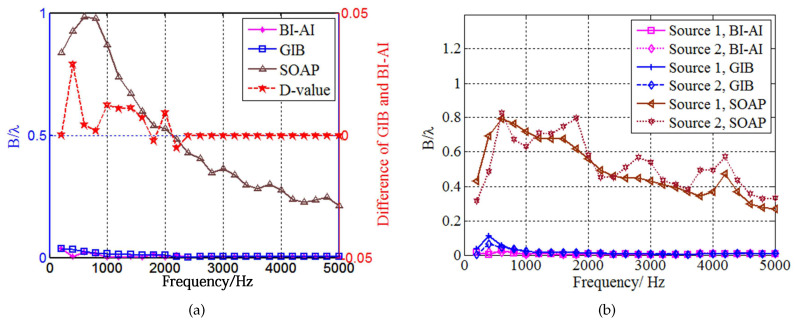
The −12 dB beam width varying radiation frequency: (**a**) one unitary monopole sound source; (**b**) two coherent monopole sound sources, the strength of source one and source one is 1 and 0.5, respectively.

Figure 6 interprets the resolution quality of three methods. SOAP outperforms conventional beamforming in the low-frequency range and it is prone to enhancing the resolution quality in the frequency range above 600 Hz. The −12 dB beamwidth of SOAP is above 0.2 wavelengths from 200 Hz to 5000 Hz. By contrast, $\ell_{1}$-GIB and the proposed method capture remarkably superior resolution quality. The improved value of the proposed method versus $\ell_{1}$-GIB is emphasized in the sub-coordinate in Figure 6a. The D-value is the −12 dB beamwidth difference of GIB and BI-AI. The proposed method performs slightly better in the low-frequency range in comparison to $\ell_{1}$-GIB. The beamwidth in Figure 6b indicates that BI-AI shapes a more stable resolution quality in a wider frequency range. BI-AI improves the accuracy and stability of sound source intensity recognition. Despite the fact that only single and coherent sources are studied in this present case, it allows us to conveniently inspect the performance of BI-AI regarding the strength estimation and the resolution quality.

## 4. Experimental Application and Discussion

**Figure 7:**
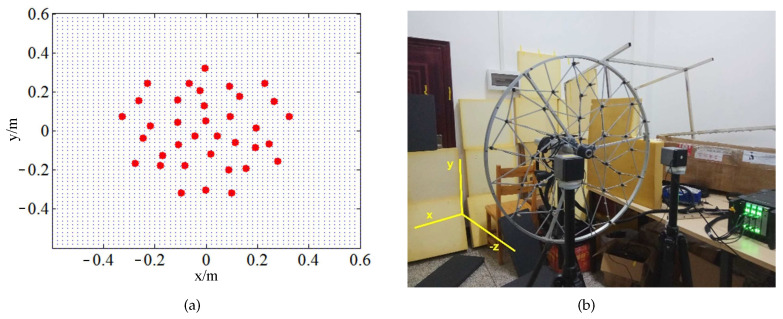
The test arrangement of experiments: (**a**) microphones array and focal points; (**b**) test site of coherent sources.

The experiments were implemented in an ordinary room. The experimental setup is arranged at the center of the room to alleviate the reverberation and coherent interference from the walls. For our experiments, the sound field generated by the loudspeakers was simultaneously acquired by HBK 36-microphone Combo Array with a 0.65-m-diameter measuring aperture, and the loudspeakers placed at 0.19 m (for NAH) and 0.6 m (for BF) respectively away from the microphone array. The sampling frequency was 16,384 Hz and the sampling time was 5 s. Figure 7 illustrates the element position of the array, the targeted focal points/equivalent sources, and the test site. The two coherent sources were placed at (−0.2, 0) m and (0.2, 0) m. The targeted focal points/equivalent sources were spotted at the grid of 1.2 m × 1.2 m regular rectangle around the origin of coordinate, parallel to the plane of array (in the x–y plane) and in the plane of source with 0.02 m spacing.

**Figure 8:**
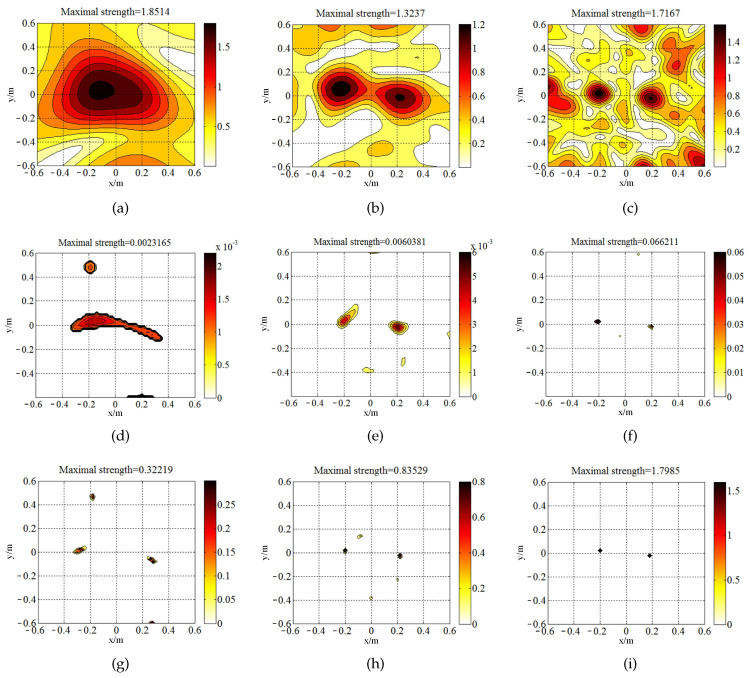
The sources reconstructed for far-field by SOAP, $\ell_{1}$-GIB, and BI-AI (with unit of $m^{4}/s^{2}$): (**a**) SOAP, 500 Hz; (**b**) SOAP, 1500 Hz; (**c**) SOAP, 3000 Hz; (**d**) $\ell_{1}$-GIB, 500 Hz; (**e**) $\ell_{1}$-GIB, 1500 Hz; (**f**) $\ell_{1}$-GIB, 3000 Hz; (**g**) BI-AI, 500 Hz; (**h**) BI-AI, 1500 Hz; (**i**) BI-AI, 3000 Hz.

As aforementioned manipulation, the eigenvalue decomposition constructed firstly an inverse problem of far-field acoustic imaging. The same as the simulation, the last component (with the largest eigenvalue) is chosen as a principal component, and the same operation could be found in [20,21]. Three methods including SOAP, $\ell_{1}$-GIB, and the proposed method identified the sound sources at a measurement distance of 0.6 m (Figure 8). At the frequency of 500 Hz, SOAP and $\ell_{1}$-GIB fail to separate the two sources. At the frequencies of 1500 Hz and 3000 Hz, the resolution quality of source imaging of BI-AI is higher than those of SOAP and $\ell_{1}$-GIB. BI-AI tends to overestimate the strength of sources (Figure 8i) since the energy of the entire radiator is represented from the spots in a restricted area. The point of beamforming here is to use the high resolution to accurately localize the hot spot of sound sources.

To interpret how the mechanism of the proposed method proceeds, the methodologies of the three methods are inspected. For statistically optimal array processing (SOAP), no iteration has been employed. SOAP charts the solution in the least mean square sense. In a practical sense, the mean square method is prone to give a flat and blurry solution, and it could narrow down the side lobes with many measurements. By contrast, $\ell_{1}$-GIB and BI-AI leverage the $\ell_{1}$ norm to enhance the sparsity.

**Figure 9:**
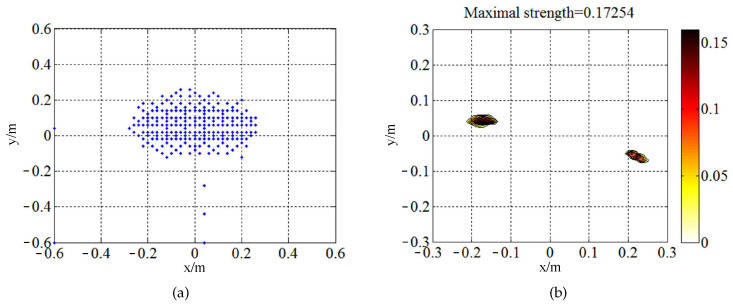
The results of adding computational grid refining at 500 Hz by wavelets method (with unit of $m^{4}/s^{2}$). (**a**) the essential points of extracted (blue dots); (**b**) the sources reconstructed in the essential region.

The same as $\ell_{1}$-GIB, BI-AI tends to deliver depressing performance in the low-frequency range, which could be explained by the increased condition number of propagation matrix G in Equation (2). According to the outputs, the interpolating wavelet method extracts the essential points of sources map of conventional beamforming to enhance the performance of BI-AI. Figure 9a shows the refined computational grid. The spacing of the grid in BF is 0.02 m. To retain the character of under-determination of the problem, the compression rate with large value is not suggested. BI-AI is newly performed in the essential region of 0.6 m × 0.6 m. The two sources in Figure 9b are closer to the real position compared to Figure 8g. Meanwhile, the pick value in (−0.2, 0.48) m disappears. The accuracy of recognition is improved because we take the source distribution into account. The wavelet decomposition can be regarded as the multilevel representation of the discrete function (the source map of CB). The fluctuations at different positions are measured by corresponding wavelet coefficients. It is the principle that wavelets can be available to carry out the multi-resolution analysis.

**Figure 10:**
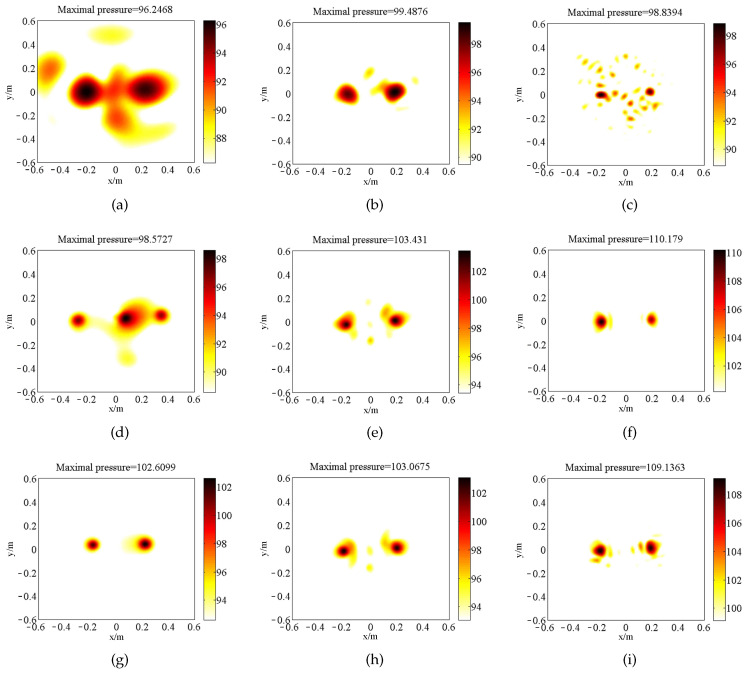
The sources reconstructed for near-field by CESM, WBH, and BI-AI (with unit of dB, 100 dB = 2 Pa): (**a**) CESM, 500 Hz; (**b**) CESM, 1500 Hz; (**c**) CESM, 3000 Hz; (**d**) WBH, 500 Hz; (**e**) WBH, 1500 Hz; (**f**) WBH, 3000 Hz; (**g**) BI-AI, 500 Hz; (**h**) BI-AI, 1500 Hz; (**i**) BI-AI, 3000 Hz.

Figure 10 illustrates that the reconstructed sound field with a holographic distance of 0.19 m. CESM delivered large main lobes at the frequencies of 500 Hz and 1500 Hz even though the reconstruction plane was closed to the source plane. At the frequency of 3000 Hz, the fake sources appeared in the presentation of CESM lead to the messy peak value of pressure around the true sources (Figure 10c). BI-AI has comparable performance with WBH at the corresponding frequency. Particularly, BI-AI reconstructs the sound field of sources on the right-hand side while WBH underestimates the right source. Meanwhile, WBH fails to reconstruct the reasonable field generated by the two sources. The experimental results are consistent with the simulated results displayed in Figure 6b, where WBH underperforms in the low-frequency range. The difference in the results of BI-AI and WBH could be attributed to the iteration strategies.

Although WBH constructs a mechanism of filtering, the wake sources via a factor of dynamic range, and the main updating strategy of WBH is still the steepest descent (SD) algorithm which is recognized as keeping the steepest character in a local area. By contrast, the fast iterative shrinkage-thresholding algorithm (FISTA) is used to solve the sub-problems of BI and each iteration of FISTA is a filtering operation. The regularization parameter λ in Equation (9) is fixed, whereas WBH searches the step length using the current gradient. Additionally, the convergence of the filter operation in WBH was not proved, whereas the Bregman iteration was strictly proved to be convergent by researchers [25].

## 5. Conclusions

The goal of this present study is to achieve robust acoustic imaging based on Bregman iteration and iterative shrinkage-thresholding algorithm. To that end, based on the $\ell_{1}$ norm minimization, the Bregman iteration method is proposed to solve the optimization problem about the sound field reconstruction and the sound source identification. The reversion from measurements to sources is recast in the form of sparse representation. Subsequently, the Bregman iteration decomposes the problem into a consequence of sub-problems; the interpolating wavelet method identifies the significant nodes and refines the computational grid to underpin the proposed method in the low-frequency range. The limitations of the proposed method are that the computation is time-consuming in a complex in situ environment and the retreat distance is difficult to determine as well.

The numerical simulation and experimental results indicate that the inverse problem in acoustic imaging could be modeled by the proposed method, and accurately solved by Bregman iteration based on the fast iterative shrinkage-thresholding algorithm. Overall, the proposed method narrows the main lobe and shows better strength and location estimation compared with conventional beamforming methods; it performed better sound field reconstruction in a wide frequency range compared with conventional equivalent source method based near-field acoustical holography. The regularization parameter could be a fixed value in the Bregman iteration process and the switch operation of the transition frequency is unnecessary by using the proposed method.

Among the three above-mentioned methods including WBH, $\ell_{1}$-GIB, and BI-AI, WBH introduces the forced filtering to acquire the sparse solution; $\ell_{1}$-GIB weights the solution by using the weighted minimum $\ell_{1}$ algorithm; and BI-AI performs the threshold contraction of the solution at each iterative step and then updates the subproblem. The numerical simulations and experiments compared the proposed method with the traditional methods in the frame of acoustic holography and beamforming, respectively. The results demonstrate that BI-AI is available and effective in the sparse facilitation of the sound source identification.

All told, on the advantages’ side, (1) the regularization parameter is constantly adjusted by the traditional methods in the process, but it is a fixed value in the Bregman iteration process; (2) the transition frequency is designed to combine CESM with WBH by [21], but this switch operation of the transition frequency is unnecessary by using the proposed method; (3) among the three methods, the solution to BI-AI is the sparsest; the maximal strength of BI-AI is bigger than $\ell_{1}$-GIB’s. Of note, the potential challenges facing the future applications of this study should be considered in light of the following limitations: (1) only the 36-channel random array was used in the experimental process, without considering the influence of the array form on the accuracy of identification; (2) Algorithmically, the lower the frequency of the source, the more time it takes to identify the source using BI-AI, due to an increase in the number of conditions of the transfer matrix in the low-frequency range; (3) The measured object in this present paper is simple. However, there are many complex models of sound sources that are transient, mobile, or rotating in the engineering practice.

The proposed method may give an alternative approach to the two-dimensional acoustic imaging of complicated sources as well as moving and rotating sources in the three-dimensional space. The adaptation of the wavelets on the three-dimensional space and the optimization of computing time for a large grid remain to be further studied.
